# What you see is not what you get anymore: a mixed-methods approach on human perception of AI-generated images

**DOI:** 10.3389/frai.2025.1707336

**Published:** 2025-11-19

**Authors:** Malte Högemann, Jonas Betke, Oliver Thomas

**Affiliations:** 1 Information Management and Business Informatics, Universität Osnabrück, Osnabrück, Germany; 2 Smart Enterprise Engineering, Deutsches Forschungszentrum für Künstliche Intelligenz GmbH Standort Niedersachsen, Osnabrück, Germany

**Keywords:** generative AI, disinformation, deepfakes, synthetic images, authenticity, photorealism, AI and society

## Abstract

The rapid development of text-to-image (TTI) models has made it increasingly difficult to distinguish between AI-generated and authentic photographs. This study explores human perception and detection capabilities regarding AI-generated images of landscapes, architecture, and interiors using a mixed-methods approach. A total of 104 participants took part in an online survey, classifying 50 images (25 real, 25 AI-generated) from five leading TTI models. Alongside their classifications, participants rated their level of confidence and provided optional justifications for their choices. A quantitative analysis revealed that participants correctly identified AI-generated images in 63.7% of cases overall and notably in only 29% of cases when FLUX.1-dev was used. The hierarchical model estimated lower odds of correct detection with increasing age, while education, gender, AI-tool use, media work, and editing experience showed no significant effects. Respective confidence scores highlight calibration issues and suggest potential overconfidence in more experienced groups. The qualitative analysis of 511 textual justifications uncovered several classic visual flaws such as geometric inconsistencies, unrealistic lighting, and semantic anomalies, while simultaneously showing a shift toward tacit judgments. Participants often characterized newer outputs as ‘too perfect’ or faintly uncanny. Therefore, this study emphasizes the need for visual literacy and regulatory mechanisms, especially in contexts susceptible to disinformation. The findings provide insights into vulnerable groups and raise awareness of the social risks posed by hyper-realistic synthetic media.

## 1 Introduction

The rapid advancement of artificial intelligence (AI) has led to significant progress in the field of image generation. Recent text-to-image (TTI) models, including Generative Adversarial Networks (GANs) and diffusion models, have achieved a level of photorealism in their output that makes it increasingly difficult to differentiate from authentic photographs (Meyer, 2022). Whilst these developments offer novel possibilities across a variety of domains, including design, media, advertising and entertainment, they also give rise to significant ethical and societal concerns. Recent studies have demonstrated that AI-generated images are increasingly being used for cultural, political, and societal misuse with implications for trust in media and democracy, including the intentional spread of disinformation, content manipulation, and character defamation (Ferrara, 2024; Marchal et al., 2024; Nightingale and Farid, 2022). A frequently cited example is that of a manipulated image depicting an explosion near the Pentagon, which was distributed via verified social media accounts and led to short-term fluctuations in financial markets (O’Sullivan and Passantino, 2023). Other viral examples of AI-generated images include Pope Francis wearing a puffer jacket by Balenciaga, Donald Trump getting arrested by police, and Vladimir Putin behind prison bars (Kamali et al., 2025; Lajka and Marcelo, 2023). Meanwhile, this deepfake phenomenon has also spread to video and audio media. In 2022, a deepfake video emerged of Volodymyr Zelenskyy, in which he appeared to call on Ukrainian troops to lay down their arms and stop fighting Russia (Roy and Roy, 2025). In January 2024, ahead of the November United States presidential election, it was estimated that tens of thousands of Democratic Party voters received AI-generated calls in Biden’s voice instructing them not to vote in the upcoming New Hampshire primaries (Barrington et al., 2025).

Despite the efforts of major technology firms to mitigate the dissemination of such content, AI-generated imagery continues to pose serious risks, particularly due to its scalability, virality, and the growing difficulty of distinguishing real from synthetic media (Wendling, 2024). On another note, this issue is further underscored by the “Dead Internet Theory,” which suggests that an increasing share of online content, especially on social media platforms, is no longer generated by humans but by AI (Walter, 2024). Emerging phenomena, such as AI influencers, who autonomously produce and tailor content to specific audiences, are accelerating this trend. Consequently, platforms are becoming saturated with machine-generated content, thereby undermining the authenticity of digital interactions and eroding trust in visual information (Walter, 2024). Muzumdar et al. (2025) also demonstrate, within the framework of the “Dead Internet Theory,” that AI can be utilized for engagement and so-called content farming. It can be used to produce clickbait articles, auto-generated blogs and to flood social media feeds, which further exacerbate concerns about authenticity and quality.

These developments highlight the necessity to analyze the challenges humans face in differentiating between authentic images and AI-generated images. With the development of more sophisticated TTI models, human perception and the cognitive ability to distinguish between real and synthetic images are gaining further scientific relevance. While previous studies focus on faces and full body images (Cooke et al., 2024; Frank et al., 2024; Kamali et al., 2025; Lüdemann et al., 2024; Meyer, 2022; Nightingale and Farid, 2022; Pocol et al., 2024), we explicitly focus on images without people. As demonstrated in the research by Kamali et al. (2024), there is a high degree of specificity in the elements that comprise AI-generated images of people. Therefore, this study explores how realistic and convincing synthetic content has become in everyday visual domains, which are increasingly used in digital communication, marketing, and media. To this end, we pose the following question:

RQ1: To what extent can individuals distinguish between real and AI-generated images, particularly in the categories of landscapes, architecture, and interiors and how well are their confidence judgments calibrated?

We aim to identify the demographic and visual factors that influence an individual’s ability to recognize AI-generated images. Understanding which groups are particularly susceptible to synthetic media, such as deepfakes, is crucial for developing targeted educational, technical, and policy-driven countermeasures. Simultaneously, analyzing which visual cues are frequently associated with AI-generated imagery can inform the design of future detection strategies and media literacy programs. Therefore, we formulate:

RQ2: How do specific TTI models and selected demographics relate to detection and miscalibration?

In order to address these questions, a two-stage experimental design was employed. A quantitative online survey was conducted to assess participants’ ability to classify real vs. AI-generated images and their individual confidence level. Open questions on the reasons for the image classification provide insights into the visual cues and model-specific artefacts that guided the participants’ decisions. This study goes beyond the aforementioned research and aims to determine not only how well humans can distinguish AI-generated images from real ones, but also whether there are calibration errors in the area of individual confidence judgments, in order to derive potential epistemic vulnerabilities from this. Therefore, the objective of the present study is twofold: firstly, to enhance our understanding of human perception in the context of synthetic media, and secondly, to provide practical strategies for raising public awareness about the risks associated with AI-generated images.

## 2 Text-to-image models: capabilities, limitations, and ethical concerns

TTI models represent a key branch of generative AI aimed at transforming natural language descriptions into coherent visual content. These models have gained significant momentum across fields such as design, advertising, and entertainment (Bie et al., 2024; Grewal et al., 2025; Hartmann et al., 2025). Their appeal lies in their ability to produce visually compelling, often photorealistic images from abstract textual prompts enhancing creativity, accelerating content production, and enabling synthetic data generation (Bie et al., 2024). Technically, TTI models are based on deep neural architectures such as Generative Adversarial Networks (GANs), diffusion models, and transformer-based systems. GANs, first introduced by Goodfellow et al. (2014), have demonstrated remarkable capabilities in generating high-resolution, photorealistic images, particularly of human faces. However, they are often limited by issues such as training instability, mode collapse and a lack of sample diversity, which can hinder their generalizability (Salimans et al., 2016). Although notable extensions such as StyleGAN2 (Karras et al., 2019) have addressed some of these limitations, GAN-based TTI approaches are increasingly being outperformed in terms of semantic alignment. Diffusion models have emerged as the dominant paradigm for image generation (Li X. et al., 2024). These models, including DALL-E 2 (Ramesh et al., 2022), Stable Diffusion (Rombach et al., 2022) and Imagen 2 (Saharia et al., 2022), operate via a gradual denoising process. This involves transforming random noise into coherent images over multiple iterations. This iterative refinement enables superior alignment with textual prompts and visual consistency. Nevertheless, diffusion models are computationally intensive and pose challenges for deployment in real-time applications or on edge devices (Shen et al., 2025). Transformer-based models, inspired by breakthroughs in natural language processing, aim to improve compositional reasoning and contextual understanding in image generation. Google’s PARTI (Yu et al., 2022) and Muse (Chang et al., 2023) are examples of autoregressive and masked transformer architectures.

Despite these advances, the images generated with TTI models exhibit persistent weaknesses, which can help to identify AI-generated images. Common failure patterns include geometrical distortions, violations of physical laws and misinterpretations of complex spatial or semantic relations as well as incorrect or unreadable text rendering (Borji, 2023; Kamali et al., 2025). Moreover, synthetic humans often display anatomical abnormalities, such as distorted hands or inconsistent facial symmetry, highlighting unresolved challenges in fine-grained generation (Borji, 2023; Kamali et al., 2025). However, recent advances in TTI models have already minimized some of these common errors. For example, OpenAI’s 4o image model produces coherent text and made advancements in photorealism with correct shadows and reflections (OpenAI, 2025). Black Forest Labs’ recent image model FLUX.1 Kontext introduces better character consistency and the possibility to modify images with simple text instructions allowing to tell stories with the same character in different settings (Batifol et al., 2025). While technical limitations are diminishing in newer models, ethical concerns in TTI models are rising. Their ability to create highly realistic but entirely fictional imagery enables misuse in the form of synthetic disinformation and deepfakes. Such content can be weaponized to spread false narratives, manipulate public opinion, or impersonate individuals in political, journalistic, or commercial contexts (Ferrara, 2024; Marchal et al., 2024). Cases of AI-generated images falsely depicting real-world events such as explosions or fabricated identities demonstrate how synthetic content can undermine public trust and even impact financial markets (Kamali et al., 2025; O’Sullivan and Passantino, 2023). Furthermore, there is a growing risk that social media platforms will be flooded with AI-generated imagery. This development could blur the boundaries between authentic and synthetic content, undermining trust in visual media and contributing to a decline in the perceived credibility of genuine information and content (Walter, 2024).

## 3 Related works

Several recent studies have examined the human ability to distinguish between AI-generated and real images, with a particular focus on facial or person-related content. For example, Kamali et al. (2025) reported an overall recognition accuracy of 76% for synthetic photographs and 74% for authentic photographs in a large-scale study comprising 749,828 observations of 50,444 participants. Their dataset included portraits, full-figure shots and group scenes generated by models such as Midjourney, Stable Diffusion and Adobe Firefly. However, no demographic data on the participants was collected, and thus, no conclusions about potentially vulnerable groups could be drawn. Nightingale and Farid (2022) conducted a series of experiments with 315 and 219 participants, asking them to classify AI-generated and real human faces. In the first experiment, participants achieved an accuracy rate of 48.2% without prior training; this increased to 59% after exposure to training material that highlighted flaws in AI-generated images, which demonstrated that awareness can be cultivated through a targeted learning strategy. Frank et al. (2024) extended this research to a broader media context in a transnational study involving 3,002 participants and multiple content types. For images, the average detection accuracy fell slightly below chance level (50%). Pocol et al. (2024) conducted a more targeted study in which 260 participants were tested on their ability to classify ten real and ten AI-generated images (produced by Stable Diffusion and DALL·E 2). The overall accuracy achieved was 61%. Participants were more accurate in identifying real images (68.5%) than synthetic images (52.6%), and no statistically significant differences were found across gender or age groups. Cooke et al. (2024) conducted a study with 1,276 participants and widened the focus from images to audio, videos and audio-visual media types. In their sample, the accuracy was 51% overall and 49.4% on just image-based content. The findings indicated that the level of accuracy was found to be lower for images of human faces (46.6%) in comparison to landscape images (54.4%). In contrast, another study by Lu et al. (2023) with 50 participants report an average accuracy rate of 64.33 and 66.37% for images of men and women but an accuracy rate of 56.50% for landscapes and 50.83% for other objects. Despite the differentiation in these two studies, AI-generated images beyond the domain of human faces, such as landscapes, architecture and objects, remain comparatively under-explored. Given their potential role in disinformation campaigns, commercial manipulation, and visual persuasion, this non-human image types warrant greater attention in future studies on the perceptual and ethical implications of synthetic media.

## 4 Methodology

To address the research questions of this study, a mixed-methods approach was adopted, integrating both quantitative and qualitative methods. This combination increases the validity of findings through methodological triangulation and offsets the limitations inherent in each individual approach (Bryman, 2006). In this context, a mixed-methods design is particularly suitable, as neither purely qualitative nor purely quantitative methods alone can adequately capture the complexity of the research questions. Quantitative analysis is essential for evaluating detection performance, confidence calibration, and statistical relationships with demographic variables. However, the qualitative component provides deeper insights into the visual cues that participants relied on to identify AI-generated images. This dual approach ensures that measurable patterns and subjective reasoning processes are both taken into account.

In April 2025, 104 German-speaking adults completed an online survey. Participants were recruited via professional networks, social media, email distribution lists and newsletters in research networks. Inclusion required age ≥18 and German language proficiency. Each person saw 50 images in random order: 25 were real photos and 25 were AI-generated (five images from each of five models). For every image, participants chose real or AI-generated and rated their confidence from 0 (not confident at all) to 100 (very confident). Participants could optionally provide a textual explanation for why they believe an image is AI-generated. Before the task, we collected demographic information such as age, gender, education, experience with AI-based image tools, frequency of AI usage, and self-assessed competence in detecting AI-generated content. Participants were not informed about the exact distribution of real and AI generated images. Participation was voluntary, consent was obtained on the landing page, and data were handled under GDPR. The structure of the survey is provided in the Supplementary materials.

Quantitatively, we used statistical methods that consider each decision and account for differences between people and images. Rather than averaging everything together, this model estimates how recognition accuracy varies by model and by participant characteristics. We evaluated discrimination, or how well confidence scores separate correct from incorrect judgments, and we evaluated calibration, or how well confidence matches reality, Qualitatively, we coded the written explanations to identify the visual cues people relied on, such as geometry, lighting, and textures.

### 4.1 Model selection

The TTI models used in the experiments were selected from the GenAI Arena, a comprehensive benchmarking framework designed to evaluate the latest image generation models. The GenAI Arena was selected because of its systematic methodology, transparent evaluation criteria and regular updates, which ensure the inclusion of the most recent and high performing models (Jiang et al., 2024). The framework combines automated performance assessments using standardized benchmark datasets with human evaluations, resulting in a robust and multidimensional evaluation of models. Key evaluation dimensions include image quality, semantic coherence and computational efficiency, providing a nuanced view of model performance in various contexts. A core criterion for inclusion in the GenAI Arena is public accessibility. Only openly available models are admitted, supporting the reproducibility of research findings and transparent model comparisons. In addition to meeting these requirements, eligible models must demonstrate strong performance across core quality benchmarks and be compatible with the GenAI Arena’s evaluation protocols (Jiang et al., 2024).

In contrast to alternative platforms, such as artificialanalysis.ai, which includes both open-source and closed-source models, but lacks methodological transparency, the GenAI Arena offers a clearly defined, reproducible framework. Unlike older benchmarks, such as the HEIM Framework (Lee et al., 2023), the GenAI Arena provides a more up-to-date basis for selecting and comparing top-performing TTI models. By providing this consistent comparison environment and emphasizing transparency, the GenAI Arena offers a scientifically validated framework for selecting models for this study (Jiang et al., 2024; TIGER-Lab, 2025).

The top five selected models based on ranking are:

1. FLUX.1-dev (Black Forest Labs, 2025)
2. Playground v2.5 (Li D. et al., 2024)
3. FLUX.1-schnell (Black Forest Labs, 2025)
4. Playground v2 (Li et al., 2023)
5. Kolors (Kolors Team, 2025)

### 4.2 Image generation

**Figure 1:**
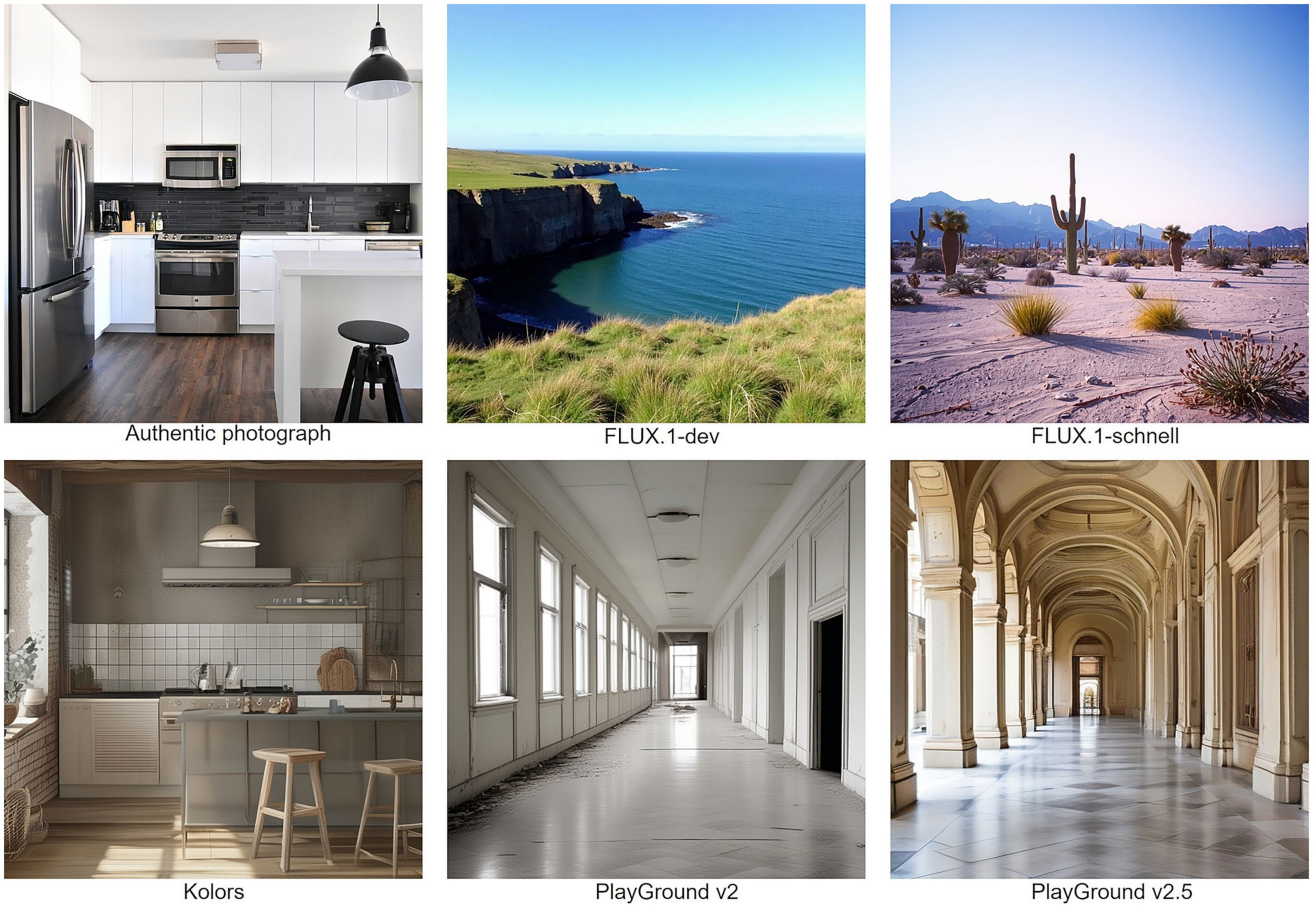
Selection of images shown in the survey (“Authentic photograph”, gray steel 3 door refrigerator near modular kitchen by Naomi Hébert, reproduced with permission from Unsplash, https://unsplash.com/photos/gray-steel-3-door-refrigerator-near-modular-kitchen-MP0bgaS_d1c. FLUX.1-dev image created using generative text-to-image AI under CC-BY-NC license, https://huggingface.co/black-forest-labs/FLUX.1-dev. FLUX.1-schnell image created using generative text-to-image AI licensed under Apache 2.0, https://huggingface.co/black-forest-labs/FLUX.1-schnell. Kolors image created using generative text-to-image AI licensed under Apache 2.0, https://huggingface.co/Kwai-Kolors/Kolors. Playground v2 image created using generative text-to-image AI; Playground v2 is licensed under the Playground v2 Community License, https://huggingface.co/playgroundai/playground-v2-1024px-aesthetic. Playground v2.5 image created using generative text-to-image AI; Playground v2.5 is licensed under the Playground v.2.5 Community License, https://huggingface.co/playgroundai/playground-v2.5-1024px-aesthetic).

Image generation was guided by structured prompts based on Liu and Chilton (2022). Prompts followed the format “A [SUBJECT] in the [STYLE]” with the goal of producing photorealistic images of landscapes, buildings, and interiors. Styles such as “high-resolution photography” and “unreal engine” were selected for their effectiveness in generating realistic outputs. Metadata-like suffixes (e.g., “IMG_87234. CR2”) were appended to some prompts to imitate filenames from real digital cameras. No images containing text were produced due to limitations in the selected models’ capabilities at the time of image generation. After generating images, we curated the 25 images used in this study. The 25 authentic photographs were sourced from Unsplash (2025) (unsplash.com) and Pixabay (2025) (pixabay.com), platforms providing royalty-free images. All selected images were published under an open license, which permits free use for scientific and academic purposes without the need for additional permission or attributions. Figure 1 shows a selection of the chosen images.

### 4.3 Quantitative analysis

Subsequent analysis includes descriptive statistics, overall and per-model classification accuracy, and comparison of detection rates between models. To account for clustering, we first quantified the intraclass correlation (ICC) at participant and image levels, deriving a corresponding design effect to quantify the loss of independent information. This assessment revealed significant stimulus-level clustering, prompting hierarchical trial-level analysis. Consequently, all confirmatory inferences were based on a mixed-effects logistic regression model with crossed random intercepts for participants and images and fixed effects for image type, generator and prespecified covariates. This specification preserves trial-level information and yields valid standard errors under the crossed structure (Gelman and Hill, 2007). Furthermore, the Brier score (Brier, 1950) was used to evaluate the accuracy of the confidence-weighted decisions made by the participants. The score ranges from 0 (perfect calibration) to 1 (maximum miscalibration), with lower values indicating better alignment between confidence and correctness. As a scoring rule for binary outcomes, it quantifies the mean squared difference between predicted probabilities and actual outcomes and is widely used in probabilistic classification tasks (Wilks, 2011). To assess the alignment between participants’ confidence levels and their actual performance, confidence calibration curves were plotted. These curves visualize the relationship between predicted confidence and observed accuracy, providing insight into systematic over- or underconfidence (Niculescu-Mizil and Caruana, 2005; Guo et al., 2017). Receiver operating characteristic (ROC) analysis with area under the curve (AUC) was also used to further support this (Fawcett, 2006).

### 4.4 Qualitative analysis

The qualitative analysis focuses on participants’ optional explanations for why they classified an image as AI-generated. To support the qualitative interpretation of the free-text responses, a word cloud was generated to highlight the most frequently mentioned terms as an exploratory tool (McNaught and Lam, 2010). Similar approaches have been adopted in related studies, including those by Lago et al. (2022) and Pocol et al. (2024). All responses are categorized according to common types of errors and visual cues based on the frameworks of Kamali et al. (2024, 2025) and Borji (2023). Categories include:

- Geometry: Unrealistic proportions, perspective errors, or dysfunctional object layouts.
- Stylistic Artifacts: Blurred textures, plastic surfaces, or digital aberrations.
- Physics: Implausible shadows, gravity-defying elements, or incorrect reflections.
- Semantics and Logic: Contextual implausible elements, illogical spatial reasoning or scene composition.
- Intuition: Gut feelings or unarticulated reasoning.

The frequency distribution of categories was visualized using a pie chart, in line with standard qualitative content analysis practices (Zhang and Wildemuth, 2009). This visualization enables a clearer understanding of which visual cues participants most frequently relied on when identifying AI-generated content, offering insights into common reasoning patterns. The five categories were used as deductive classes, and respective mentions were assigned to them according to the subclasses. Two coders independently coded a stratified subset of the data (approximately 20% or 100 comments). After the initial double-coding pass, the coders met to compare their decisions, resolve disagreements, and refine the definitions and examples of the codes. Overall, coding was straightforward within the deductive categories. The main source of ambiguity arose from longer comments that warranted multiple categories. Inter-coder reliability on this subset was quantified using Krippendorff’s alpha (Krippendorff, 2018) yielding an alpha value of 0.8601, indicating substantial agreement. After reaching a consensus on the scheme and procedures, the two coders divided and single-coded the remaining comments.

## 5 Results

**Figure 2:**
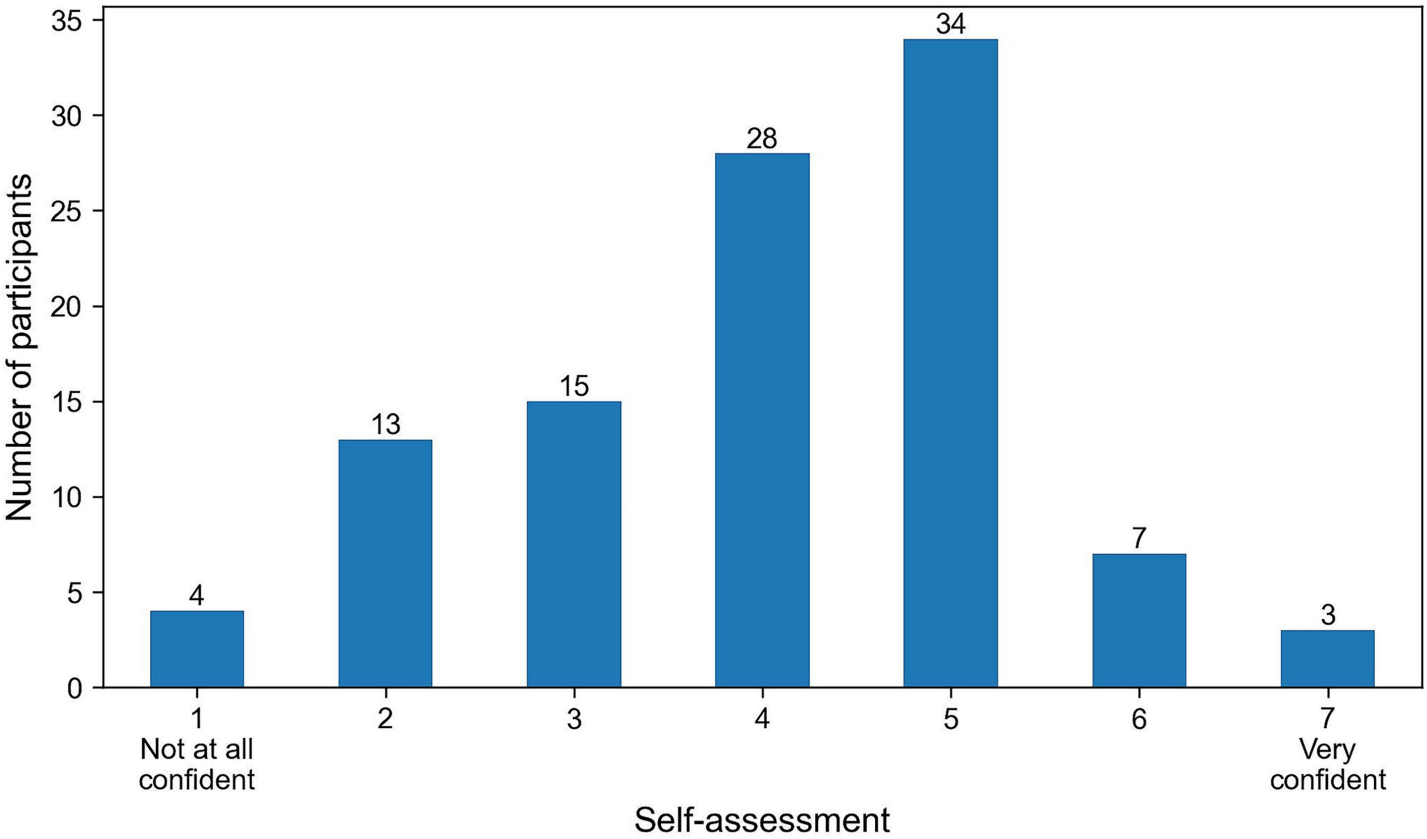
Self-assessment in the recognition of AI-generated images.

A total of 104 participants took part in the online study and fully completed it. 74 aborted or incomplete questionnaires were not included. This led to a total of 5,200 observations of AI-generated images and real images. The largest age group was 25–34 years old (*n* = 34), followed by 18-24-year-olds (*n* = 31). 18 participants were aged 35–44 years, while 11 and 10 participants were in the 45–54 and 55–64 age brackets, respectively. A total of 67 people who identify as male and 37 who identify as female took part in the survey. No respondents selected the ‘diverse’ option or chose to withhold their gender. In terms of educational background, most participants had advanced secondary or higher qualifications. Specifically, 48 respondents reported having an equivalent to the High school diploma, 29 held a bachelor’s degree and 15 held a master’s degree. One participant indicated having a lower secondary school certificate, and 11 had an intermediate secondary school certificate. No participants reported having completed a doctorate or having no formal education. When asked about their prior experience with AI-based image tools, 62 participants stated that they had experience, while 42 reported having none. Regarding the usage of AI tools usage patterns varied: 44 participants reported regular use, 46 used such tools occasionally, and 14 did not use AI-based tools at all. Only 15 participants indicated that they work professionally or privately with visual media while 89 respondents reported no regular engagement in this area. Finally, participants were asked to self-assess their ability to distinguish between real and AI-generated images using a 7-point Likert scale. Most responses were clustered around 4 and 5, resulting in an average confidence rating of 4.03 (SD = 1.27). Fewer participants rated their ability as either very high or very low (Figure 2).

### 5.1 Quantitative results

**Figure 3:**
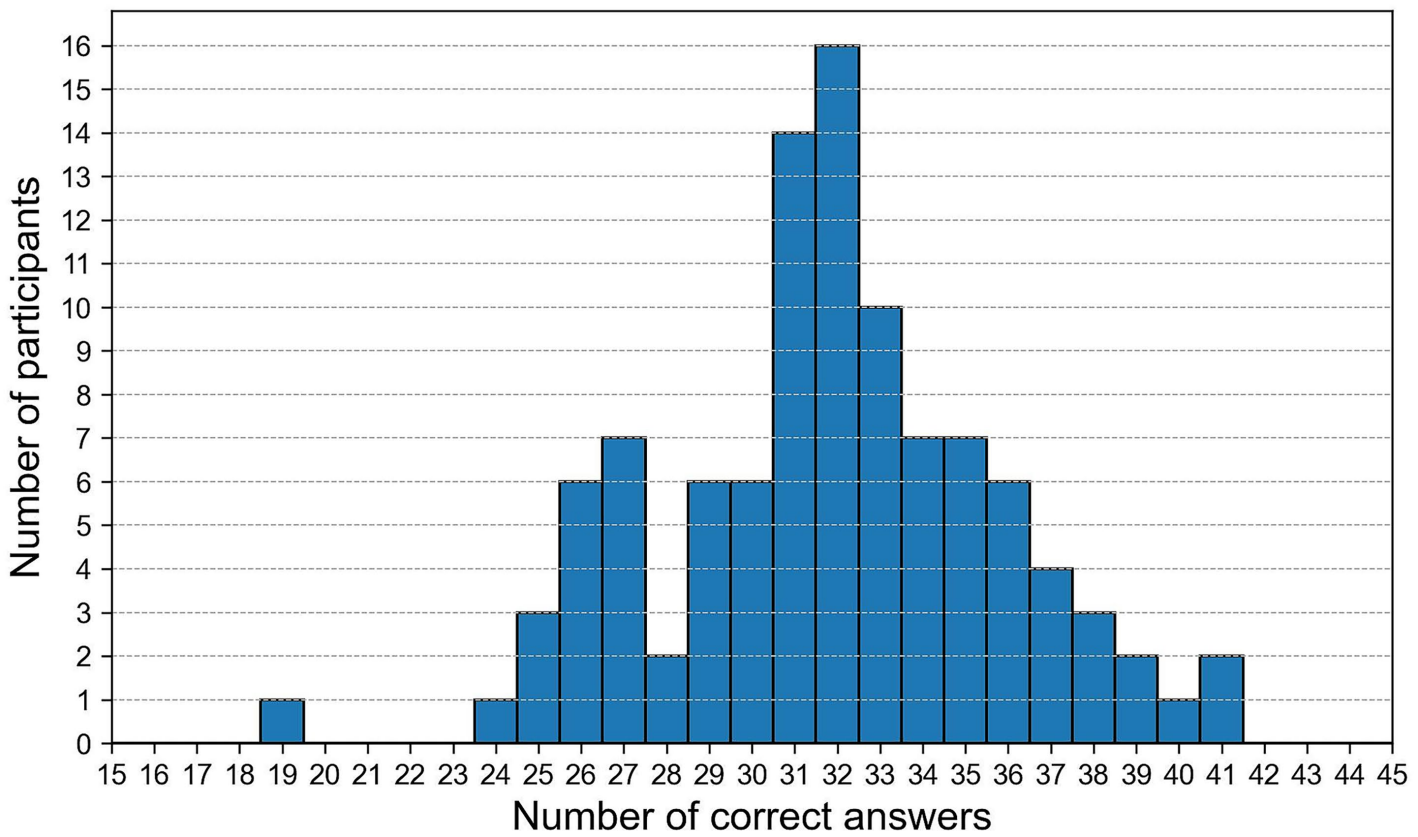
Distribution of the total number of correct answers per participant.

Participants correctly identified an average of 17 AI-generated images, with a standard deviation of approximately 3. The minimum number of correct classifications was six, while the maximum was 23. The 25th and 75th percentiles were 15.75 and 19 respectively, suggesting that the majority of participants performed within this range. In contrast, the results for authentic photographs show slightly lower average recognition performance. The mean number of correct identifications was 14.78 and a standard deviation of 3.27. Performance ranged from six to 24 correct responses, with a 25th percentile of 13 and a 75th percentile of 17. Considering combined recognition performance (the sum of correctly identified real and AI-generated images) the mean total score was 31.83, with a standard deviation of 3.97. The range extended from a minimum of 19 to a maximum of 41 correct identifications. The 25th percentile was 29.75 and the 75th percentile was 34, indicating that most participants clustered around this central range with one significant outlier (Figure 3). Based on descriptive statistics it can be seen that younger participants tended to achieve higher recognition scores.: Participants in the 18–24 and 25–34 age categories attained average scores of 32.55 and 32.94, respectively, while those in the 55–64 age group demonstrated lower average performance (M = 29.40). With regard to gender, male participants achieved a higher mean performance than their female counterparts (M = 32.43 vs. M = 30.73). Participants in possession of a master’s degree achieved the highest average score (M = 32.60), followed by those with a bachelor’s degree (M = 32.10), while individuals with lower secondary education performed lowest (M = 30.00). Experience with AI-based image tools has also demonstrated a positive tendency. Participants who reported such experiences demonstrated a higher mean performance (M = 32.44) in comparison to those who did not (M = 30.93). Regular and occasional users demonstrated higher average scores (M = 32.11–32.26) compared to those who reported never using such tools (M = 29.50). Participants with professional experience in visual media showed marginally higher performance (M = 32.20) compared to those without (M = 31.76). While not statistically significant, a positive trend was observed between participants’ self-assessed competence in recognizing AI-generated content and their actual performance. The mean recognition scores exhibited a gradual increase from 27.25 among those who rated themselves as least confident to 34.00 among those who rated themselves as most confident.

Table 1 presents the mean recognition rate, standard deviation, and mean confidence rating for each of the five TTI models included in the study. The findings indicate considerable heterogeneity across models: while images generated by the Kolors model were correctly identified in 86.73% of cases on average, those produced by FLUX.1-dev achieved a substantially lower accuracy rate of 29.04%. In contrast, the confidence rating of 65.42 for this model is only marginally lower than that of the other models.

**Table 1:** Mean accuracy, standard deviation and mean confidence per TTI model (5 images × 104 participants = 520 trials per model).

Model	Mean Accuracy Rate	Std. Deviation Accuracy Rate	Mean Confidence Rating
FLUX.1-dev	29.04%	17.73	65.42
Playground v2.5	68.27%	19.70	66.69
FLUX.1-schnell	74.23%	10.51	69.01
Playground v2	82.69%	12.87	71.24
Kolors	86.73%	6.45	71.17

Prior to testing, we estimated the ICC, which yielded the following results: Participant = 0.007 and Image = 0.243, based on a one-way random-effects decomposition. Given the study design (50 trials per participant and 104 judgments per image), these values imply a design effect of approximately 26.34 and an effective sample size of approximately 197 out of 5,200 trials. Accordingly, all confirmatory inferences were conducted at the trial level using a mixed-effects logistic model with crossed random intercepts for participants and images (Gelman and Hill, 2007). In doing so, we fit a hierarchical logistic mixed-effects model to predict trial-level correctness (1/0) using a Bernoulli–logit mixed-effects regression. We employed the Bayesian estimation with Hamiltonian Monte Carlo (No-U-Turn Sampler) via PyMC v15.4.1, using Bambi v0.15.0 (Capretto et al., 2022) and ArviZ v0.22.0 (Kumar et al., 2019). We chose 4 chains, 2,000 warmup (tuning) iterations and 2,000 posterior draws per chain (8,000 post-warmup draws total), with target accept = 0.99 and random seed = 42 (Betancourt, 2017; Hoffman and Gelman, 2014). We used weakly informative Bambi defaults on the logit scale: the intercept had a Normal (0, 3.5355) prior and fixed-effect slopes had Normal (0, 5.0) priors. For the hierarchical terms, the group-level standard deviations were HalfNormal (3.5355), and the random intercept deviations for participants and images were Normal (0, σ_group), with σ_group drawn from the respective HalfNormal prior (Capretto et al., 2022). Convergence diagnostics were satisfactory for all fixed and group-level parameters (max R^ = 1.01; minimum effective sample sizes: bulk = 523, tail = 945). No divergent transitions occurred, the fraction of iterations at the tree-depth limit was negligible (0.013%), and E-BFMI values were > 0.3 for all chains (≈ 0.67–0.74), indicating adequate energy mixing (Betancourt, 2017; Kumar et al., 2019; Vehtari et al., 2021).

Using posterior draws, we computed pairwise model contrasts on the log-odds scale, reported as odds ratios (OR) with 95% credible intervals and Holm-adjusted two-sided tail probabilities. With FLUX.1-dev as the baseline, participants were substantially less likely to classify FLUX.1-dev images correctly compared to Kolors (OR = 0.05, 95% CI [0.01, 0.17], Holm = 0.0025), Playground v2 (OR = 0.07, 95% CI [0.01, 0.21], Holm = 0.0045), and FLUX.1-schnell (OR = 0.14, 95% CI [0.03, 0.46], Holm = 0.034). The difference vs. Playground v2.5 (OR = 0.17, 95% CI [0.03, 0.58]) did not survive multiplicity correction (Holm = 0.07) and should be treated as exploratory. No other pairwise comparisons among non-baseline models were statistically reliable after correction (all 95% CIs included 1; all Holm ≥ 0.60). The posterior distribution indicated a small and uncertain advantage for AI-generated images (log-odds = 0.54, 95% highest density interval (HDI) [−0.19, 1.29]; OR = 1.71, 95% HDI [0.83, 3.62]). Age showed a robust negative association with accuracy (per category: log odds = −0.13, 95% HDI [−0.23, −0.03]); OR = 0.88, 95% HDI [0.80, 0.97]). The HDIs of all other covariates included zero, suggesting no clear effects. Posterior predictive checks indicated an excellent absolute and relative fit. The observed overall accuracy (0.637) was virtually identical to the posterior predictive median (0.637; 95% CI [0.621, 0.652]), with a symmetric Bayesian *p*-value of approximately 0.998, suggesting there is no discrepancy for this statistic. At the image level, the posterior predictive means closely tracked the observed accuracies (r ≈ 1.00), indicating that the model effectively captures cross-image difficulty (Kumar et al., 2019; Gelman et al., 2013).

**Figure 4:**
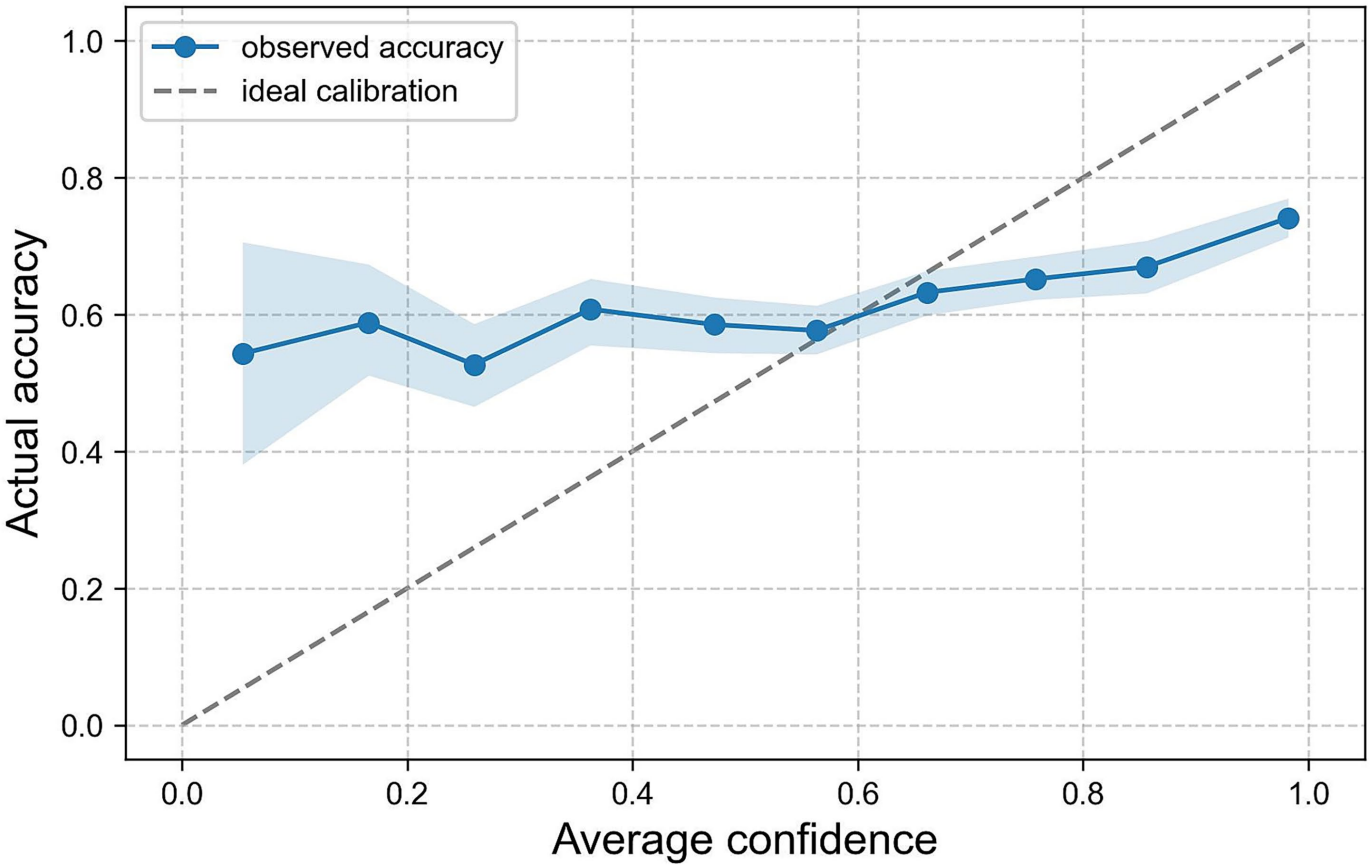
Confidence calibration curve aggregated on all models and participants with 95% CIs.

A calibration analysis was conducted to assess the alignment between participants’ subjective confidence and their actual recognition performance. The confidence ratings, which were provided on a scale from 0 to 100 for each classification decision, were normalized and grouped into 10 bins of equal width. For each bin, the mean confidence level was plotted against the corresponding empirical accuracy level, yielding a calibration curve (Figure 4). This curve shows the extent to which participants’ confidence levels reflected their actual accuracy. Ideal calibration would align with the diagonal reference line, where subjective confidence matches observed accuracy perfectly. The observed curve deviates from the ideal, particularly in the lower and higher confidence range. This discrepancy can be quantified using the Expected Calibration Error (ECE), which summarizes the average absolute difference between confidence and accuracy across bins. ECE was 0.142 with 10 bins and 0.146 with 20 bins, indicating conclusions robust to bin choice and suggesting a moderate degree of miscalibration in participants’ confidence judgements.

**Figure 5:**
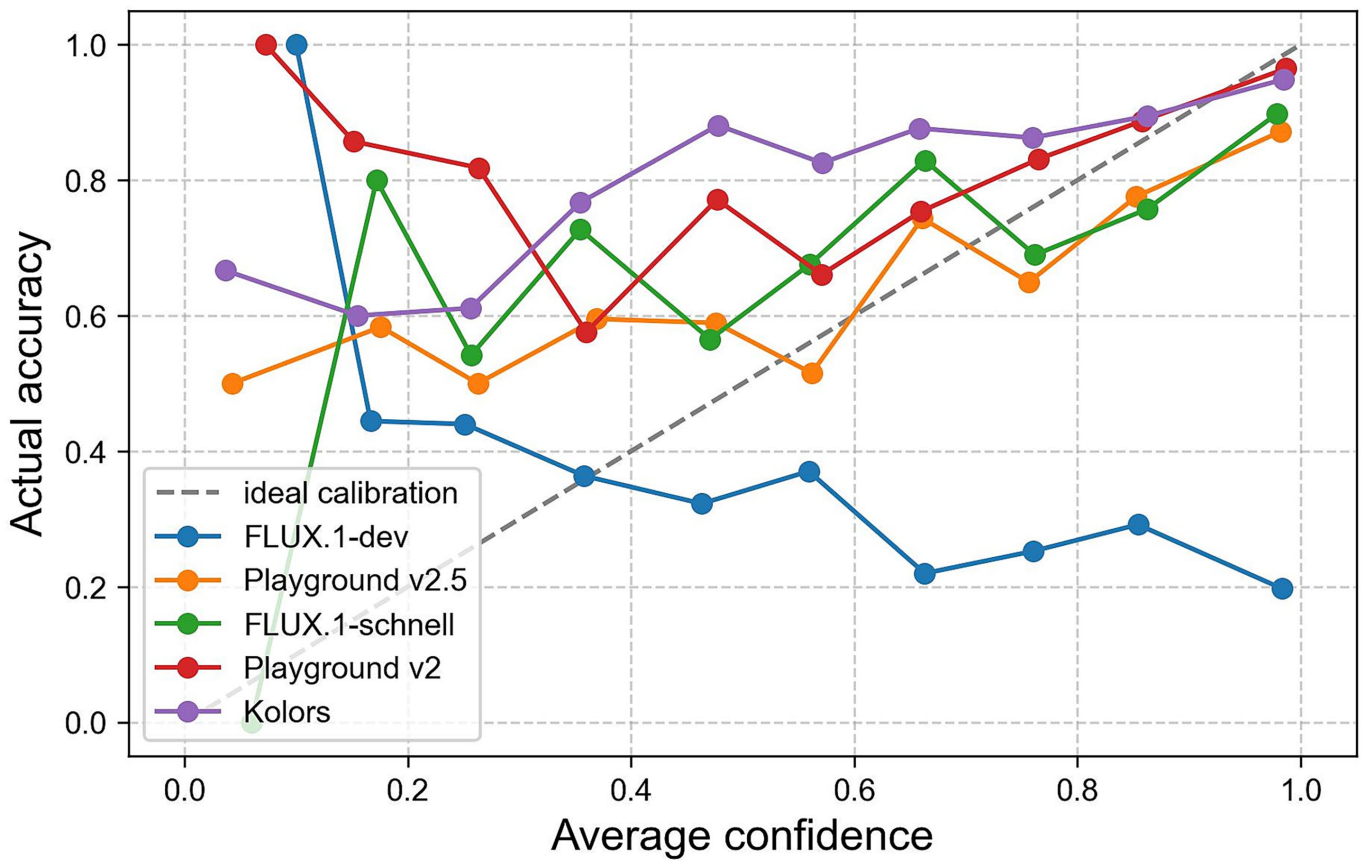
Confidence calibration curve aggregated on each model.

In Figure 5, calibration curves are presented for each of the five models separately, with participants’ average confidence being compared with their actual classification accuracy across bins. Four of the models demonstrate a moderate or good alignment between confidence and accuracy in higher confidence ranks, while exhibiting underconfidence in lower ranks. FLUX.1-dev, however, demonstrates a significant discrepancy, with accuracy diminishing with higher confidence, suggesting a substantial degree of overconfidence. This assumption is substantiated by an ECE of 0.396 [95% CI, 0.356, 0.440] which is notably higher than that of the other models. In contrast, Playground v2.5 (ECE = 0.119 [95% CI, 0.085, 0.161]), Playground v2 (ECE = 0.127 [95% CI, 0.1, 0.161]), and FLUX.1-schnell (ECE = 0.135 [95% CI, 0.100, 0.171]) demonstrate more consistent calibration. Kolors, despite having the highest overall recognition rates, shows marginally diminished stability in calibration (ECE = 0.174 [95% CI, 0.145, 0.206]), particularly within the mid-confidence range. Figure 5 with the respective 95% CIs can be found in the Supplementary materials. We complemented this with ROC and the area under the curve AUC computed from trial-level confidence as the score (Fawcett, 2006). Overall, discrimination was modest (AUC = 0.57, 95% CI [0.55, 0.59]). By image type, the AUC was higher for AI images (0.61, [0.59, 0.63]) than for real images (0.53, [0.50, 0.55]). Among the TTI models, confidence reliably discriminated between correct and incorrect images for Playground v2 (AUC = 0.69), Kolors (AUC = 0.67), Playground v2.5 (AUC = 0.65), and FLUX.1-schnell (AUC = 0.63). However, FLUX.1-dev showed below-chance discrimination (AUC = 0.42, [0.36, 0.48]), indicating overconfidence errors within this model.

Lastly, we evaluate the correlation between participants’ perceived confidence and their actual recognition performance. To achieve this, Brier scores (Brier, 1950; Wilks, 2011) were calculated for various demographic and experiential subgroups. The overall Brier score was 0.259, indicating moderate calibration accuracy across the sample. Age-related differences emerged, with the 18–24 age group (Brier = 0.238) exhibiting better calibration than older groups, particularly the 55–64 age group (0.317). Regarding gender, male participants demonstrated slightly more accurate calibration (0.245) than female participants (0.285). Educational background also appeared to play a role, with participants who received an intermediate level of education demonstrating better calibration (Brier = 0.230; 0.242) than those with higher qualifications (Brier = 0.282; 0.279). Those without prior experience in AI-based image tools showed better alignment between confidence and accuracy (0.228) than those with experience (0.280). A similar pattern emerged in AI tool usage: individuals who had never used AI tools achieved the lowest Brier score (0.207), compared to those who used them rarely (0.262) or regularly (0.272). Regarding professional experience with visual media, those without such a background were more accurately calibrated (0.253) than those with it (0.296). Finally, an analysis by self-assessed competence revealed that participants with lower confidence levels (ratings 2–3) exhibited the lowest Brier scores (0.217 and 0.197), while those with very low or very high self-assessments showed poorer calibration (e.g., rating 1: 0.287; rating 7: 0.376).

### 5.2 Qualitative results

**Figure 6:**
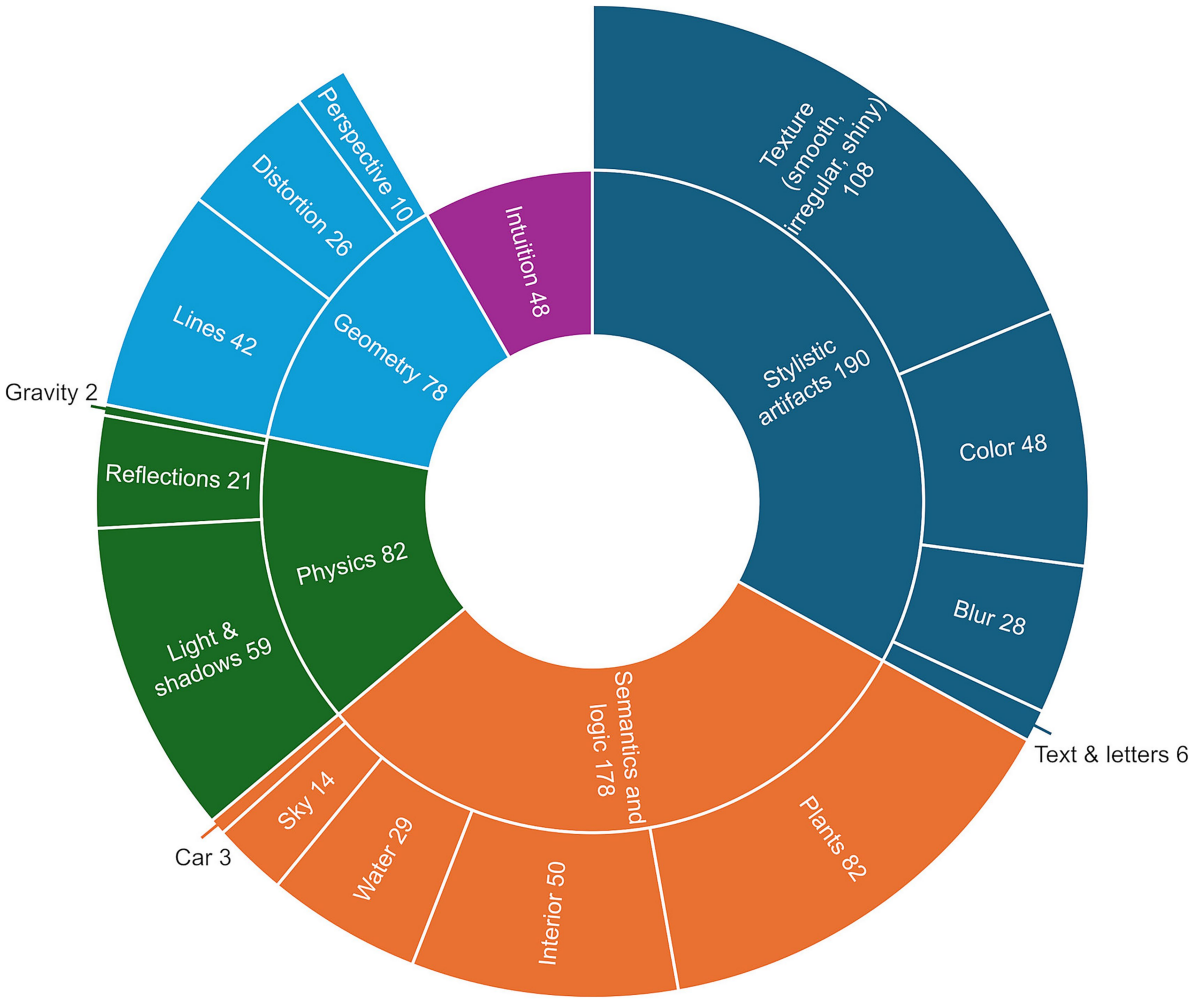
Sunburst diagram on qualitative analysis of cues.

To enable an in-depth qualitative analysis of participants’ assessments of images, we examined all free-text comments in which participants explained why they believed an image to be AI-generated. Although optional explanations were collected for every image, regardless of whether it was real or synthetic, only comments referring to AI-generated images were included in the analysis. In total, 511 valid comments were evaluated, ranging from single words to detailed, multi-sentence explanations. Following a qualitative review and coding process, a total of 576 distinct mentions were extracted, as some comments contained multiple identifiable aspects. These mentions were then categorized thematically and structured according to a hierarchical classification scheme inspired by Borji (2023) and Kamali et al. (2025). To explore and visualize the most common terms, we generated a word cloud before reviewing (Supplementary material) and complemented it with a sunburst chart after reviewing (Figure 6). Frequently cited aspects in the word cloud include details, texture, lighting, and colors, which suggests that many participants relied on general visual cues not specific to the image contents. Other terms, such as “heaven,” “mirror,” “blurred,” and “distorted,” indicate attention to image-specific anomalies. Mentions of objects such as tables, plants, and fire extinguishers show that semantic elements and details in certain objects also played a role in the participants’ judgments.

Categorizing 576 coded mentions from participants’ explanations revealed a diverse range of visual features referenced when classifying images as AI-generated, which complements most of the findings in the word cloud. The largest group of references was categorized as stylistic artefacts (*n* = 190), with texture-related aspects (e.g., smoothness, irregular surfaces or overly polished materials) being the most frequently noted (*n* = 108), followed by color (*n* = 48) and blur (*n* = 28). The second-largest group of mentions was assigned to semantics and logic (*n* = 178), including observations related to plants (*n* = 82), interior elements such as furniture or decor (*n* = 50), and representations of water (*n* = 29) and sky (*n* = 14). This indicates that object placement and contextual relationships were commonly referenced in the classification process. Mentions categorized under physics (*n* = 82) primarily referred to light and shadow conditions (*n* = 59) and reflections (*n* = 21), while the geometry category (*n* = 78) included comments on lines (*n* = 42), distortion (*n* = 26) and perspective (*n* = 10). Additionally, 48 comments were grouped under the intuition category, capturing responses in which participants described a general impression or subjective sense that the image was artificial without specifying concrete visual elements. Beyond exploratory visualizations with existing taxonomies, we found that a large part of comments across all models referred more to subtle errors and clues, as they focus on stylistic irregularities like to smooth or polished materials, oversaturated or picturesque colors or too evenly arranged objects. Overall FLUX.1-dev received 35 comments of which several comments referred to an uncanny, unnatural feeling or a too perfect look: “The lanes look too clean”; “The street looks artificial”; “lines that are too perfect”; “The whole style reminds me of AI. I cannot explain it exactly”; “everything perfectly aligned”; “The image somehow makes me feel uncomfortable and gives me a headache.”; “The floor has a strange texture. Parts of the image look too perfect in some areas”; “Too perfectly arranged”; It looks extremely unnatural”; “Has kind of an uncanny valley vibe.”

On the other side rather obvious and classic errors still prevail in the other models, especially in the areas of physics, logic and geometry: “The reflections in the window do not reflect a palm tree”; “The reflection in the window looks very clear, but at the same time you can still see through the window very clearly”; “The shadow angles do not match at all”; “Serious errors in things such as proportion, sharpness, shape, etc.”; “Building appears structurally flawed.” One response sums up the situation, pointing out that several errors only become apparent upon closer inspection.: “It looks extremely unnatural. Shapes are distorted, things that should be straight are crooked. The longer you look, the more you find.” We provide an additional codebook in the Supplementary data with the respective categories, subcodes and anchor examples from the data.

## 6 Discussion

While most previous studies focus on human faces and bodies (Frank et al., 2024; Kamali et al., 2025; Lüdemann et al., 2024; Meyer, 2022; Nightingale and Farid, 2022; Pocol et al., 2024), we focused on landscapes, architecture and interior to show that images on the internet and especially on social media may no longer be identified with certainty as authentic or fake. Regarding RQ1, our findings align with several previous studies, revealing an overall recognition accuracy of 63.7%. Notably, one model (FLUX.1-dev) emerged as an outlier which was confirmed by the mixed-effects analysis. Overall, participants performed significantly above chance level, demonstrating a moderate reliable ability to distinguish between real and AI-generated images. Compared to other studies, we used more sophisticated, state-of-the-art models that are less error-prone than models like DALL-E2 or older Stable Diffusion models, which were mostly employed in previous studies. Still, a similar outcome in terms of overall accuracy was achieved, which could suggest that people are becoming better or more careful at detecting anomalies in AI-generated images. Directly comparing the accuracy and respective confidence rating per model (Table 1) shows that, except for FLUX.1-dev, participants estimated their average confidence to be lower than their actual accuracy. Our findings in the confidence calibration curves (Figure 4) further support the idea of increased caution. The overall accuracy in the lower reported confidence brackets (0–0.6) fluctuated between 53 and 60%, showing that participants may underestimate their abilities. This is clearer when comparing the models (Figure 5), where underconfidence is shown up to the 0.9 bracket for Playground v2 and Kolors. In the higher confidence brackets (0.7–1.0), it can be seen that, except for FLUX.1-dev, accuracy and confidence almost align. This reveals that, most of the time, participants were right when they were confident. In the case of FLUX.1-dev, however, the opposite is true. The higher the confidence, the worse the accuracy, with a discrepancy of almost 0.8 in the highest bracket and an ECE of 0.396. The findings are further supported by the ROC/AUC. This suggests that this particular model opens avenues for epistemic vulnerability. Findings regarding the Brier score show that the calibration between confidence and actual accuracy is better for groups of people who tend to have less experience in using AI, image processing or working with visual media than for the group with more experience. These findings suggest that individuals with more experience in AI or visual media may exhibit an overconfidence bias when evaluating AI-generated imagery, leading to a weaker alignment between their perceived certainty and actual performance. In contrast, less experienced participants appear to demonstrate more cautious judgment, resulting in a better-calibrated relationship between confidence and correctness. The qualitative analysis confirms that, when identifying AI-generated images, participants frequently referred to well-documented visual artifacts, such as issues with texture, lighting, and geometry. However, models like FLUX.1-dev demonstrate that some of these indicators are becoming less reliable. Overall, images of FLUX.1-dev received only 35 comments in which participants rarely mentioned classic flaws related to shadows, perspective, or reflections. This observation aligns with recent advancements in image generation technologies, which have minimized many previously common artifacts. They focused either on specific objects in the images or mentioned that some aspects seemed too perfect to be real. While classic errors prevailed in several images of other models, they got more subtle, which suggests that the way we perceive AI-generated images is shifting, with people now relying more on their instincts and finding images that seem too perfect uncanny. These patterns align with socio-technical accounts of the impact of AI on human interpretive agency (Di Plinio, 2025). Sustained exposure to AI-mediated environments can gradually shift decision-making heuristics away from the detection of discrete artefacts and towards uncanny or ‘too-perfect’ regularities, thereby reshaping perceived agency (Di Plinio, 2025). These developments underscore the increasing difficulty of detecting synthetic content and highlight the need for ongoing research aligned with the rapidly evolving state of the art. Overall, the analysis of confidence scores suggests that participants focused on identifying specific artifacts or relied on intuition indicating that an image was AI-generated, which were less prevalent in FLUX.1-dev and consequently might felt too safe. This could lead to rather harmless consequences when AI-generated images are used to draw attention on websites and social media or for unfair advertising of non-existent landscapes or buildings. A greater danger could come from the use of harmful images to manipulate groups of people, especially in politics, including spreading fear.

The demographic analysis shows that participants in higher age brackets demonstrate a statistically significant worse accuracy performance, which supports similar findings by Lüdemann et al. (2024). This indicates a need for training or intervention for particularly prone populations to minimize negative effects. On the basis of descriptive stats male participants, individuals with higher formal education, those with experience in AI-based image tools, and those who rated their skills as higher on a 7-point scale all demonstrated higher recognition accuracy on average. Overall, we therefore recommend further research to identify particularly vulnerable user groups in the context of AI-generated content. Tailored training and educational materials could strengthen these users’ detection capabilities. Evidence from Nightingale and Farid (2022) shows that training can significantly improve classification accuracy. Furthermore, previous studies have found a link between media literacy and susceptibility to misinformation, emphasizing the importance of media education in reducing risks (Frank et al., 2024; Hwang et al., 2021; Jeong et al., 2012). With a view to Brier scores and self-assessment, our findings suggest that even participants with higher expertise might feel too confident and struggle to reliably detect AI-generated images, indicating that training efforts and media literacy must be seen as a collective cultural task, not only an individual competence.

Although progress is being made in the field of automated AI-generated image detection (Konstantinidou et al., 2025), with good results being achieved in test environments, these approaches are not yet suitable for application to online content (Corvi et al., 2023; Karageorgiou et al., 2024). While these technical solutions might play an important role in detecting AI-generated images, we argue that a complementary, socio-technical approach is essential. Specifically, human-centered strategies such as media literacy programs, targeted training, and awareness campaigns can help individuals better identify synthetic content and reduce susceptibility to deception. In addition to technical and educational measures, regulatory frameworks are beginning to address the challenges posed by synthetic media. The EU AI Act, for example, includes provisions that require clear labeling of AI-generated content, including images, to ensure transparency for end-users (European Union, 2024). Complementing this, researchers have called for the establishment of ethical guidelines for the development and distribution of TTI models (Al-Kfairy et al., 2024; Ferrara, 2024; Nightingale and Farid, 2022).

We present a compilation of visual anomalies and inconsistencies that participants identified in their free-text responses. We categorized these using a content analysis approach (Zhang and Wildemuth, 2009) and established classification frameworks by Borji (2023) and Kamali et al. (2025). Our analysis shows that participants most frequently referenced stylistic artifacts and semantic or logical inconsistencies. Next were physical violations and geometric errors. Least frequently referenced were intuitive or gut-feeling based judgments. An important insight from our data is that participants most often explicitly referenced observable flaws or uncertainties within the images rather than relying on general assumptions, indicating a relatively high level of critical engagement with the visual content. However, our results also suggest that traditional categories become increasingly less applicable, especially in the case of more sophisticated models, such as FLUX.1-dev. As image generation technology continues to advance rapidly, previously well-established taxonomies may no longer capture the full range of current model behaviors. In this context, continuously tested and refined taxonomies and classification frameworks may serve as valuable foundations for the design of aforementioned educational interventions, enabling structured guidance on typical image flaws and cognitive detection cues. Therefore, given our finding that higher AI and media literacy is associated with inflated confidence at a given level of accuracy, as well as the practical reality of rapidly evolving generative models where yesterday’s artifacts quickly become obsolete, we conclude that training and UI should emphasize calibration feedback, adversarial examples, and uncertainty elicitation rather than categorical accuracy alone.

Limitations of this study include the sample size of participants and unequal distribution in demographic data, as the sample is a German speaking convenience sample, which limits generalizability and the found effects should be treated exploratory on broader population. A further limitation of the sample is that we showed five images per model. However, we were still able to show that there is statistical significance in the comparison of accuracy between the respective models. Nevertheless, we would like to point out that aspects relating to epistemic vulnerability in the case of FLUX.1-dev should be considered exploratory. It cannot be completely ruled out that random effects within the sample played a role in the case of the five images. Furthermore, we did not include paid models but rather focused on open-source models with APIs that are accessible to everyone and could be utilized without major effort even on local devices. Therefore, models such as Imagen 4 or the TTI model of GPT-4o were excluded despite their superior performance evaluations on artificialanalysis.ai. Another limitation of this study is that participants were given a clear task and unlimited time. In real-life scenarios, such as visiting a website or scrolling through social media, the accuracy rate may be lower because people pay less attention to potential errors in images. Cooke et al. (2024) addressed this factor in their study by developing a design that emulated online platforms and utilized a variety of stimuli. Similarly demonstrated by Kamali et al. (2025), implementing a time restraint results in lower accuracy scores. This design could be tested in future research.

## 7 Conclusion

This study examines individuals’ ability to detect AI-generated images in photorealistic landscapes, architecture, and interiors. While participants achieved moderate overall recognition accuracy, substantial differences were observed among models, with certain advanced systems, such as FLUX.1-dev, yielding significantly lower detection rates. Qualitative analysis further revealed that participants often relied on stylistic artifacts and semantic inconsistencies to make judgments. Though these cues might be less reliable due to recent model advancements and more subtle and gut-feeling heuristics could become more relevant. Although technical detection methods will continue to be vital for identifying synthetic content, we argue that a complementary socio-technical approach is also necessary. Specifically, human-centered strategies, such as media literacy programs, targeted training, and awareness campaigns, can empower individuals to recognize AI-generated imagery more effectively and reduce their susceptibility to deception. Well-defined, empirically tested taxonomies might provide a basis for these educational efforts. In parallel, regulatory frameworks are beginning to respond to the societal challenges posed by synthetic media. The EU AI Act already mandates the labeling of AI-generated content to ensure transparency and minimize risk of disinformation. Beyond compliance, there is a need for ethical guidelines to govern the development and distribution of TTI models in the future to maintain a balance between development and progress while reducing negative aspects.

As TTI models continue to evolve, the boundaries between reality and fiction online are blurring. The flood of hyper realistic, AI-generated visuals threatens to erode trust in digital imagery and raises pressing questions about the future of human-authored content. If we fail to act, we must ask: will genuine visual content on the internet gradually cease to exist?

## Data Availability

The datasets presented in this study can be found in online repositories. The names of the repository/repositories and accession number(s) can be found at: https://myshare.uni-osnabrueck.de/d/aebfe400633945e08717/.
