# Supplementary material for: What you see is not what you get anymore: a mixed-methods approach on human perception of AI-generated images
Source: Front Artif Intell. 2025 Nov 19;8:1707336. doi: 10.3389/frai.2025.1707336 (PMC12672458; doi:10.3389/frai.2025.1707336)
Supplement: Supplementary file 1 [file Data_Sheet_1.pdf]

Codebook

Based on Borji (2023). Categories include:

- **Geometry**: Unrealistic proportions, perspective errors, or dysfunctional object layouts.
- **Stylistic Artifacts**: Blurred textures, plastic surfaces, or digital aberrations.
- **Physics**: Implausible shadows, gravity-defying elements, or incorrect reflections.
- **Semantics and Logic**: Contextual implausible elements, illogical spatial reasoning or scene composition.
- **Intuition**: Gut feelings or unarticulated reasoning.

**Category**	**Subcode**	**Anchor examples from data**
Geometry	Distortion / warping	“Either this is another bold design or AI distortion. Everything looks crooked and skewed, and what kind of dishwasher is that? :D”
Geometry	Lines / linearity	“Some lines are not straight”
Geometry	Perspective	“Rear right door too small.”<br>“Proportions of the polar bear.”
Stylistic artifacts	Texture (smooth/irregular/shiny)	“Extremely shiny and polished, without any distinctive surfaces.<br>“Texture from grain and path.”
Stylistic artifacts	Color	“The colors are too strong.”<br>“Unnatural colors, everything is equally saturated.”
Stylistic artifacts	Blur	“A hut can be seen in the window, which looks unreal and blurred”<br>“Nature appears spongy and out of focus.”
Stylistic artifacts	stylistic	“Either this is a real image with strong stylization or AI.”<br>“Overall, it looks unnatural; the house/lighting conditions look too perfect.”
Physics	Light & shadows	“The lighting and shadows are strange.”
Physics	Reflections	“The reflections in the window do not reflect a palm tree; the leaves in the reflection do not match.“
Physics	Gravity	“Violations of physical laws.”

Semantics and logic	Plants / vegetation	The bushes in the foreground look strange (as if individual blades of grass were floating in the air).
Semantics and logic	Interior / furnishing	“The flame in the fireplace has unclear textures, the vase at the bottom left appears two-dimensional; unrealistic object in the middle in front of the sofa.”
Semantics and logic	Water / waves	“The way the water behaves seems strange to me. The lighting of the sea seems unreal and the textures are unclear.”
Semantics and logic	Sky	“The sky in the picture looks as if a sandstorm must be raging, but the photo is very clear.”<br>“The sky looks strange, the same color as the sand.”
Intuition	(no subcode)	“I don't know either, but that's what my gut feeling tells me.”<br>“Seems strange to me”
