# Supplementary material for: What you see is not what you get anymore: a mixed-methods approach on human perception of AI-generated images
Source: Front Artif Intell. 2025 Nov 19;8:1707336. doi: 10.3389/frai.2025.1707336 (PMC12672458; doi:10.3389/frai.2025.1707336)
Supplement: Supplementary file 2 [file Data_Sheet_2.pdf]

Survey on the Detection of AI-Generated Images

Thank you for taking part in this study!
This survey is part of an academic study and examines how well people can distinguish between real and AI-generated images. Your participation helps us better understand human perception and the limits of current AI technologies.

Procedure

- You will view **50 images** in total from the categories **landscapes, buildings, and interiors**.
- For **each image**, indicate whether you believe it is **real** or **AI-generated**.
- Then **rate your confidence** in that judgment on a scale from **0 (not confident at all)** to **100 (very confident)**.
- You may add a comment on why you believe an image is AI-generated
- In this survey there is **no time limit**; please proceed at your own pace.

Notes

- Your responses are **anonymous** and used **solely for scientific purposes**.
- There are **no right or wrong answers**—we are interested in how you perceive the images.

Estimated duration: **20–30 minutes**.

When you are ready, click **Next** to begin.

By choosing **Next**, you confirm that you:

- are **18 years or older**;
- **voluntarily** agree to take part in this research study;
- understand that your responses will be used **for scientific purposes** (e.g., analysis, publication, teaching);
- understand that your data will be stored **anonymously** and handled in accordance with applicable data-protection laws;
- may **stop the survey at any time** and **withdraw without penalty**, simply by closing the browser window.

Thank you for your support!

Demographic Questions

1) How old are you?*
Please select one:

- Under 18
- 18–24
- 25–34
- 35–44
- 45–54
- 55–64
- 65 or older

2) What is your gender?*
Please select one:

- Male
- Female
- Diverse / Non-binary
- Prefer not to say

3) What is the highest level of education you have completed?*
Please select one:

- No school-leaving certificate
- Lower secondary education (e.g., Hauptschulabschluss)
- Intermediate secondary education (e.g., Realschulabschluss)
- Higher education entrance qualification (e.g., Abitur / Fachhochschulreife)
- Bachelor’s degree (or equivalent)
- Master’s degree (or equivalent)
- Doctorate/PhD

4) Do you have experience with AI-based image tools?*
Please select one:

- Yes
- No

5) How often do yo use AI-based tools?*
Please select one:

- Regularly
- Occasionally
- Never

6) Do you work with visual media professionally or privately (e.g., photography, graphic design)?*
Please select one:

- Yes
- No

7) How confident are you in your ability to distinguish real images from AI-generated images?*
Please select one:

- 1 — Not confident at all
- 2
- 3
- 4
- 5
- 6
- 7 — Very confident

*Required items
