# Supplementary figures and images for: What you see is not what you get anymore: a mixed-methods approach on human perception of AI-generated images
Source: Front Artif Intell. 2025 Nov 19;8:1707336. doi: 10.3389/frai.2025.1707336 (PMC12672458; doi:10.3389/frai.2025.1707336)

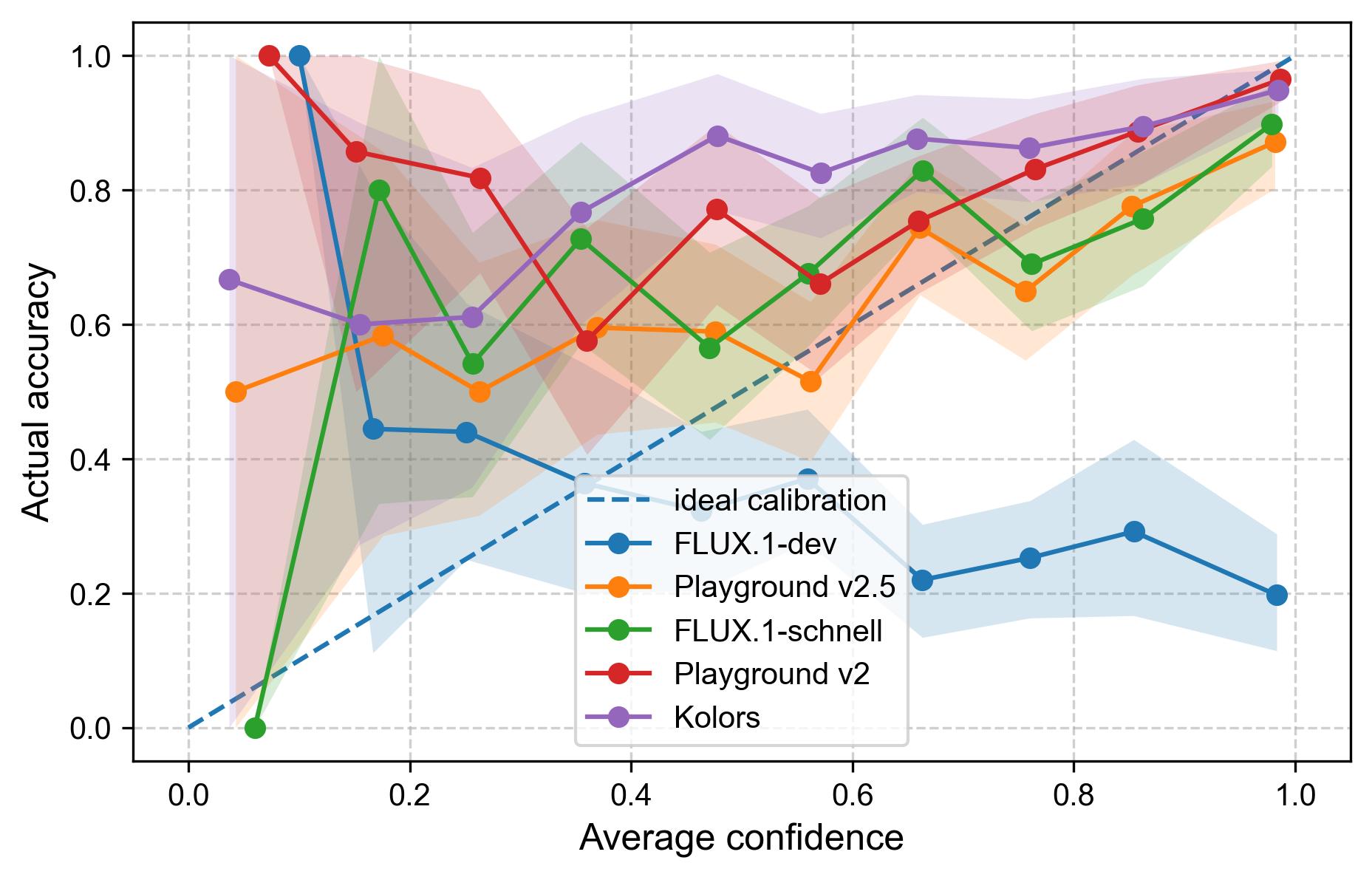

Supplement: Supplementary file 3 [file Image_1.jpeg]

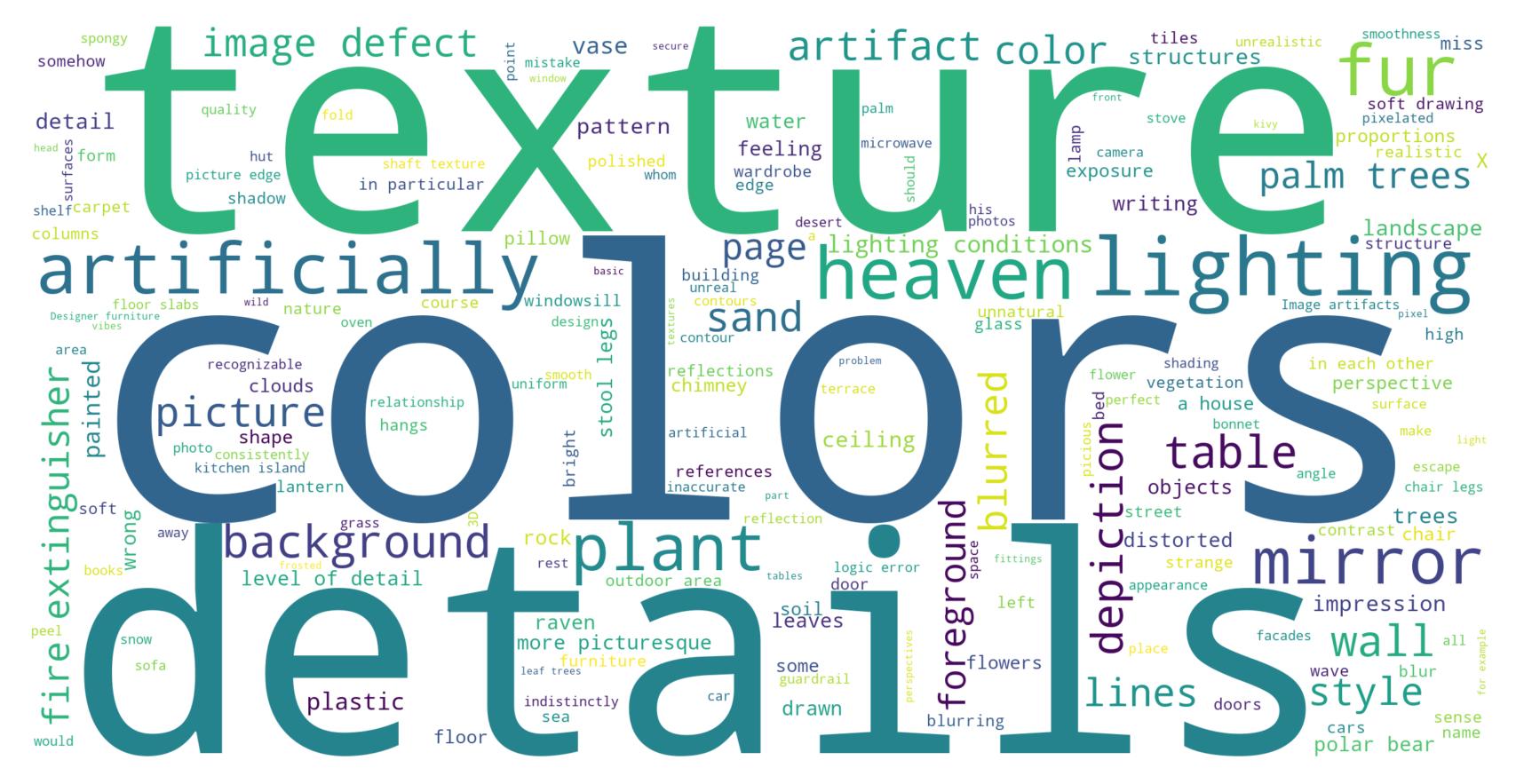

Supplement: Supplementary file 4 [file Image_2.jpg]
